# Structural Stability and Disorder Level of Moderately Reduced Paper-like Graphene Oxide Investigated with Micro-Raman Analysis

**DOI:** 10.3390/ma17040877

**Published:** 2024-02-14

**Authors:** Karol Adam Janulewicz, Tomasz Fok, Bartosz Bartosewicz, Andrzej Bartnik, Henryk Fiedorowicz, Przemysław Wachulak

**Affiliations:** Institute of Optoelectronics, Military University of Technology, Kaliskiego 2, 00-908 Warsaw, Poland; karoljanulewicz51@gmail.com (K.A.J.); tomasz.fok@wat.edu.pl (T.F.); bartosz.bartosewicz@wat.edu.pl (B.B.); andrzej.bartnik@wat.edu.pl (A.B.); henryk.fiedorowicz@wat.edu.pl (H.F.)

**Keywords:** graphene oxide paper, micro-Raman analysis, structural disorder

## Abstract

This paper discusses the results of the micro-Raman analysis performed on paper-like graphene oxide (GO) samples consisting of many functionalised graphene layers and annealed at moderate temperatures (≤500 °C) under vacuum conditions (p ≃ 10^−4^ mbar). The analysis of the standalone samples revealed that the obtained material is characterised by a noticeable disorder level but still stays below the commonly accepted threshold of high or total disorder. GO formed in a simple way showed two spectral bands above 1650 cm^−1^ recorded very rarely or not at all and their origin has been discussed in detail. The results also confirmed the metastable character of multilayer GO after the annealing process at moderate temperatures as the C/O ratio was kept between 2 and 3 and the spectral features were stable within the annealing temperature range.

## 1. Introduction

Graphene oxide (GO) has attracted attention as an input material for the facile production of graphene with the potential to replace the mechanical exfoliation method with thermal or chemical reduction [1,2,3]. Surprisingly, GO itself has appeared to be interesting for many industrial applications such as those in electronics [4], energy-related ones [5,6,7] or filtering membranes in a form of GO laminate for desalinisation [8]. The material is in practice dominantly formed as single- or few-layer graphene oxide (slGO/flGO), which are analogous to slG/flG of graphene. The samples of a thickness in the range between 0.1 µm and 10 µm are a noticeably rarer object of investigation in the physico-chemical context. Such samples conserve or even strengthen the extremely good mechanical and reasonable electrical properties of the material. For this reason, there exists very strong but rather technological interest. The “thick samples” of a thickness ≥ 1 µm are frequently termed as GO paper or paper-like GO and can be considered as a sort of bulk (or 3D) material with an inexactly defined nature of interaction between the intermittently stacked layers due to the massive presence of the hydrogen bonding triggered by water and the existence of many edges. The functional groups between the basal planes show a strong tendency to self-arrangement. Water is either trapped during the forming process (Hummers’ method) or comes from the environmental humidity but most frequently both sources contribute [9]. It should be stressed that the structural properties of such a material are strongly dependent on details of the forming procedure and can, in the extreme case, differ even after repeating the same production process. Controlling, or at least understanding, such multilayer structures of GO is important for the aforementioned applications. However, this is still far from being fully explored, and the offered explanations are sometimes contradictory. The interest in the material is understandably mainly focussed on the material’s mechanical and electrical properties. While the physics and properties of single- or few-layer graphene (slG/flG) [10,11,12] as well as those of slGO/flGO [13,14,15] seem to be reasonably understood, the data related to the paper-like or very thick multilayer graphene oxide (mlGO) are limited. Such a thick material is less predictable and its complex structure opens different ordering/disordering possibilities by edges and randomised stacking, frequently with wrinkled layers [9,16]. It increases enormously the number of structural variation paths. From now on we will use the term mlGO in the context of the paper-like thick material.

It was found in [17] that mlGO is a metastable material that at a quasi-equilibrium approaches a nearly constant C/O ratio accompanied by a reduction in the epoxide content and an increase in the hydroxyl amount. Our samples confirmed this finding when investigated using the NEXAFS (near-edge X-ray absorption spectroscopy) and XPS (X-ray photo-electron spectroscopy) techniques [18]. There was also a suggestion based on the results of numerical modelling that the structural and chemical changes are initiated by availability of hydrogen contained in the water molecules [17,19,20]. The reduction process offers another external stimuli for modification of the structure by temperature and possible mechanical tensions. A dominant part of the reports on Raman spectroscopy of mlGO focusses on samples of thickness ≤100 nm, and the material in the form of paper-like GO is under-represented in the available reports.

Here, we present an additional data set of structural information obtained by applying the micro-Raman analysis to thick samples (mlGO of a thickness ≃ 1 µm). Table 1 presented below defines the influence of the production method (oxidation and annealing) on the most important parameters in our analysis by comparing them with those selected from other reports.

The micro-Raman method, being a useful source of information on material structure and composition order, is applied to add some information regarding the structure of our specific free-standing sample. The specific character of the samples resulted from the applied formation method.

## 2. Materials and Methods

Chemical method of GO production relying on oxidation and the following reduction was proposed as early as 1958 [24]. The procedure has been modified and improved (e.g., Tour’s method [1]) but always includes the phases of harsh oxidation and the subsequent reduction process to remove or limit the concentration of the functional groups and minimise the scale of the introduced defects. Water was always present in the cleaning procedures. Our free-standing samples were formed by short-time embedding a copper mesh (standard TEM grid) in a water suspension of GO platelets prepared according to the improved Hummers’ method [1,24]. The water trapped in the mesh openings contained an abundance of immersed GO platelets. It seems justified to consider the following drying procedure as a sort of gravity-caused sedimentation with a material confined within a water layer by surface tension. Hence, the situation resembles closely a sort of diagenesis—the process of a chemical and physical reaction between water and material (in this case a carbonaceous one), originally considered in geological formations but on a different time scale [25]. The samples were dried in the free airspace, and as a consequence they definitely contained a significant amount of intercalated or sealed water. Moreover, the significant length of the drying process (about 24 h) could be favourable for the possible chemical reactions in the material. Five of six samples were annealed under moderate heating and the micro-Raman analysis was conducted directly after cooling them down. A detailed description of all applied technological procedures was given in Ref. [18].

**Figure 1:**
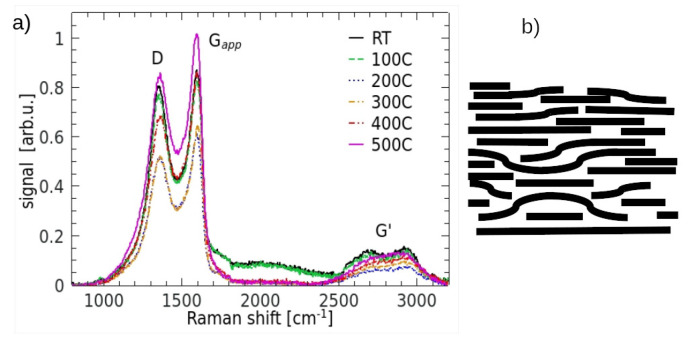
(**a**) Full Raman spectra of mlGO recorded for different annealing temperatures; (**b**) exemplary (arbitrary) schematic illustration of the disordered flake segment.

The conducted micro-Raman analysis covered the spectral interval between 800 cm^−1^ and 3200 cm^−1^. A collection of the Raman spectra of all differently annealed samples is shown in Figure 1a and an exemplary representation of the possible structure of our sample is illustrated in Figure 1b. The sample set consisted of six elements and five of them were annealed for half an hour under vacuum conditions (∼10^−4^ mbar) at temperatures between 100 °C and 500 °C with a step of 100 °C. The sixth one was not annealed and is denoted as RT. The micro-Raman measurements were carried out with a commercial system consisting of the Renishaw InVia Raman microscope equipped with an EMCCD detector. The Raman signal was generated with laser radiation at a wavelength of 532 nm (power of 2.5 mW). The laser beam was delivered to the sample through a 20×/0.4 objective giving the light spot on a sample of ≲2 µm in diameter. The wavelength of the instrument was calibrated using an internal silicon wafer with the spectrum centred at 520.5 cm^−1^. Raman spectra, presented in this paper, were acquired by averaging performed on 150 independent measurements.

## 3. Results

There is still an open problem relating to how far the structure-related quantification methods developed dominantly for single- or few-layer graphene and GO are valid for the samples of graphene oxide of a significant thickness. The comparative methods frequently applying highly oriented pyrolitic graphite (HOPG), i.e., a 3D-form of carbon, as a reference suggest that the methods developed for few-layer GO could be also applied to multilayered GO (mlGO) structures treated as a strongly defective or disordered material. For this reason, we decided to use the available methods of the micro-Raman analysis of flGO/mlGO (e.g., [15,26,27]) without a systematic investigation of the thickness dependence. However, the obtained results should be very carefully compared or extrapolated from those obtained with well-ordered carbon materials as references. Intercalated functional groups with their bonds to the basal planes and to the edges as well as those between the functionals themselves constitute an additional complication in the analysis of mlGO structure. It can be easily concluded from Figure 1a that our Raman spectrum closely resembles that of graphene chemically exfoliated from graphite oxide and presented, e.g., in Ref. [16] or that of natural microcrystalline graphite reported in Ref. [28], especially as its second-order spectrum (*G*’ sometimes denoted as 2*D* band) is concerned. An example of the possible complex morphology resulting from random stacking is illustrated in Figure 1b and such a structure clearly offers the possibility of trapping functionals or their components, the first of which is water.

**Figure 2:**
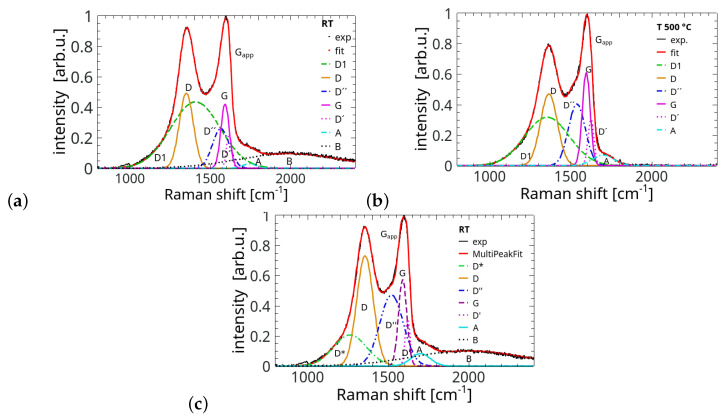
Deconvoluted *D* and *G* bands of graphene oxide paper at two different temperatures: (**a**) a room temperature (RT) and (**b**) annealed at 500 °C; (**c**) the same bands after a different (more typical) form of slightly lower quality deconvolution with the separate band $D^{*}$. The deconvolution process was performed with a goodness of fit characterised by the test value of $R^{2}\geq$ 0.999 and $\chi^{2}$ between 0.10 and 0.25.

Characteristically, the main parts of the spectrum presented in Figure 1a show an intensity relation of the spectral features typical for flGO. The $G_{app}$ band is stronger than the *D* band. The band $G^{\prime }$ containing overtones and/or a combination of the lines present in the *D*-*G* range has a form of two small bumps of comparable intensity in the area between 2500 cm^−1^ and 3200 cm^−1^. The term $G_{app}$ applies to the right intense lobe in the first-order spectrum with the peak close to 1600 cm^−1^. According to the suggestion in Ref. [29], this feature results from the contribution of both the actual *G* line and the disorder-induced $D^{\prime }$ component. Both have been separately used in the deconvolution process, as presented in Figure 2. The observed merging (overlapping) of both components is considered an indication of a remarkable disorder level in graphitic materials [12]. It has also other consequences. The careful inspection of the fitting parameters showed a significant level (10–20%) of errors in the case of weaker and narrower bands *G* and $D^{\prime }$. These errors were transferred on the parameters applied in the determination of the disorder level, specifically, $I_{G}$, $A_{G}$, ${I^{\prime }}_{D}$ and ${A^{\prime }}_{D}$. In all presented figures we used the error bars when they were larger than the size of the measurement points and did not disturb the figure’s clarity. Actually, in the case of GO, the $G_{app}$ peak’s strength and form are influenced also by the strong line $D^{\prime \prime }$ not taken into account, for example, in the analysis presented in Ref. [29] and applied to slGO and flGO. The origin of the $D^{\prime \prime }$ line is interpreted in the literature in different ways, but its intensity increase to a noticeable level with temperature during the reduction process (compare Figure 2a and Figure 2b) reasonably supports the suggestions pointing to the interstitial defects, as the annealing process causes the interlayer space to be noticeably reduced. On the other hand, the weak increase in the Raman shift of the *G* band (but still below 1600 cm^−1^) implies that we were still within the first stage of the amorphisation trajectory defined in Ref. [30]. The position of the band *G* slightly above 1590 cm^−1^ and a value of the ratio of the bands integrated intensities $A_{D}$/$A_{G}$ positioned between 1.0 and 1.7 suggest that our samples are in the middle way towards nanocrystalline graphite. Otherwise, the peak position of the *G* band should show significant down-conversion. Deep amorphisation seems to be very unlikely as the amorphisation “fingerprints” suggested, for example, in [31] and based on the relation between the peak coordinates of the lines *G* and *D* were clearly missed in our case. Moreover, Merlen et al. presented an excellent and comprehensive work [32] on the charaterisation of the defective aromatic carbon solids by the analysis based apparently on the parameters extracted without a specific fitting procedure, which made the results more realistic [33]. The analysis was based on the classical functional dependence between the position and the width of the *G* band. Our data perfectly matched the presented results, confirming that our samples were really in the middle on the way towards nano-crystalline graphite [32,33].

Analysis of the available spectra and quantification of some results bring another not fully answered question. The characteristic trends in the behaviour of the deconvolved spectral components for each amorphisation phase have been listed in Ref. [34] and it was generally recommended there to use the peak intensity ratio $I_{D}/I_{G}$ together with a width of the bands to retrieve the structural information in the case of a large disorder. The idea has been later extended to the use of integrated intensity (band areas $A_{i}$) instead of the peak intensities [34,35,36,37]. Following this recommendation and assuming a significant disorder level, we deconvolved the first-order part (*D*-*G*) of the spectrum to estimate the necessary parameters. It should be stressed that in contrast to a very popular fitting for slG and flG based on different line-shape broadening mechanisms and the fit goodness (see, e.g., [26]), we have applied a Gaussian shape. Apart from the fact that in our attempt the best Lorentzian fit was of distinctly lower quality ($R^{2}\;≃$ 0.96 in contrast to $R^{2}>$ 0.99 for Gaussian), there is a question of physical reasoning for the choice. Assuming a significant level of disorder in our samples, we preferred rather the inhomogeneous broadening mechanism of the lines/bands and this is evidently better characterised by the Gaussian shape [10,38]. The weighting factor W(q) in Saito’s Equation (7) [10] does not change the Gaussian character of the curve. It was also reported in [38] that in the case of significant disorder two options of the fitting shapes are used in practice, a mix of Lorentzian and Breit–Wigner–Fano (BWF) lines or only the Gaussian one; we opted for the second variant. Moreover, it is well known that an irregular structure (disorder) including self-organised functionals introduces inhomogeneously distributed tension (strain) and we think that it noticeably, although with different intensity, contributes to the inhomogeneous mechanism of line broadening.

Seven Gaussian components were used to deconvolve the spectrum. The results for the room temperature sample (RT) and that annealed at 500 °C are shown in Figure 2. The components resulting from deconvolution were denoted in the standard way as D1, *D*, $D^{\prime \prime }$, *G*, $D^{\prime }$, T1 and T2. Here, D1 was used for a broad band centred at ≃1400 cm^−1^ (at the RT sample) as a substitute for usually presented weaker $D^{*}$ band. This type of fitting was applied in [39,40] for carbon materials. As suggested earlier, we consider the applied forming method of the samples as a sort of quick sedimentation. The micro-Raman analysis of natural carbonaceous material is usually used in paleogeology to find specific spectral signatures treated as geothermometers. A carbonaceous material at low metamorphic temperatures (<320 °C) and advanced diagenesis indicates an increased level of disorder evidenced, e.g., by a more complex composition of the band accompanying the standard *D* band placed at ≃1360 cm^−1^ [25]. This is another reason for the deconvolution with the broad D1 band in place of the typically used $D^{*}$ band. *D* and *G* bands are the classical features in the Raman spectra of an imperfect carbonaceous material. The *D* band originates in the defected areas of the structure as the defects break the lattice symmetry cancelling prohibition of the transitions connected to so-called breathing in-plane oscillations. The $D^{\prime }$ band is closely connected to the *D* band and also is triggered by the structural defects. The *G* band is the result of $E_{2g}$ in-plane stretching oscillations of the carbon pairs.

It is worth noting that the spectra of the sample prepared at RT and that annealed at 100 °C had a nearly identical shape and both differed noticeably from the remainder of the set (Figure 1a). Interestingly, such a quasi-identity was also observed in the NEXAFS results for the samples annealed at the same temperatures [18]. Here, a broad component centred about 2000 cm^−1^ and denoted as *B* band is distinctly visible only in these two samples. The disappearance of practically the whole *B* band with heating at 200 °C perfectly matches the removal characteristics of the intercalated water [9,41]. The-close-to zero remainders of this component are also noticeable at higher annealing temperatures. Water itself shows a relatively weak and broad line in the Raman spectrum positioned at about 1640 cm^−1^, and is considered the only liquid water-related signature originating from molecular vibration in the bending mode [42,43]. Some reports suggested significant differences between the isotropic and anisotropic spectra of water [44,45] but still the band labelled as *B* is too strong (≃12% of the $G_{app}$ intensity) and too broad to be caused only by a vibrational spectrum of water or the second-order spectra and combined bands. Unfortunately, the spectral range between 3200 cm^−1^ and 3500 cm^−1^ with a very strong water signature was unavailable in the experiment to give a clear answer to the question. Due to this uncertainty, we also considered the presence of short (≤10 atoms) linear carbon chains and polyynes (C_*n*_H_2_ or -C≡C-) suspended in water with the ends fixed to the basal planes as a source of this kind of spectrum. The first of the considered compounds shows the Raman-active modes (vibrational stretching mode) at multiple frequencies between 1264 cm^−1^ and 2098 cm^−1^ [46] while the polyynes deliver some spectral components around 2100 cm^−1^ [47]. We assumed that such compounds are created during harsh oxidation in a liquid environment as repored earlier [47]. Hummers’ method used in the sample preparation corresponds well to such a characteristic. After water removal, the compounds could react with carbon and other functionals using the available hydrogen bonds.

Analysis of the absorbance spectra of an mlGO sample presented in Ref. [9] implied increased concentrations of ketone and ester carbonyl derivatives, a conclusion which is based on the reduction in the intensity of the OH-related vibrational modes. Our deconvolved spectra visible in Figure 2 show the presence of a small lob (denoted as *A*) between 1670 and 1730 cm^−1^ at all applied temperatures. The feature is very rarely seen in the recorded spectra and its origin can be debated. Such a feature was observed previously in the Raman spectra of graphene-like materials [27,48] as a peak at about 1700 cm^−1^ and of intensity equal to 2–6% of that of the corresponding $G_{app}$ peak. The band disappeared during the annealing process and was ascribed to the presence of the combination modes. On the other hand, there were reports on the signatures of electronic excitation and double-resonance present in the spectral range between 1650 cm^−1^ and 1850 cm^−1^ [49,50]. These effects are also intensely considered in connection to the control of the stacking order. However, it still cannot be an account for the existence of such a broad feature as that in the spectrum of our samples. The *A* peak in our spectrum showed an intensity of 8% in relation to $G_{app}$ and its intensity stayed within a margin of 1% during the whole annealing process. As a consequence, we tend to ascribe the feature rather to the in-plane carbonyl (C=O) and ring stretching vibrations present in quinones, esters, aldehydes, carboxylic acids and ketones [51] than to the combination mode. The latter should be visibly modified by the reduction process and our *A* band is exceptionally stable within the applied temperature range. The carboxylic acid that is the main product of material/functionals interaction with water decomposes only in the temperatures exceeding 500 °C. Moreover, carbonyl esters convert under temperatures higher than 200 °C into carboxylic acids and ketones. The latter also decompose above 500 °C. Water constitutes a crucial factor in these processes. It was suggested that cyclic ether and carbonyl groups can be generated by the reaction between two H_2_O molecules with two dangling carbon bonds, while an encounter of C-OH and C-H generates a water molecule [17]. However, cyclic ether is rather chemically unstable due to its structure containing built-in elements that cause tension. The presented scenario of the *A* band origin is not the ultimate one as we did not verify the behaviour of some bonds connected to the considered compounds by IR spectroscopy of our samples. For the sake of completeness, one has to consider in detail the aforementioned effects of electronic excitation and double-resonance with the bands appearing in the spectral range of interest. The other investigation techniques such as IR and Raman spectroscopy together with the NEXAFS would definitely be helpful in finding these solutions.

The main reasons for the use of spectral deconvolution with some additional features such as *A*, *B* and enhanced (broadened) D1 was also the fact that many of the mentioned chemical compounds involved in the described processes have weak spectral bands between 1100 and 1500 cm^−1^. Here, it was tacitly assumed that D1 includes the weaker component $D^{*}$ typically present in the spectra of slGO/flGO. As such a modification has very serious consequences, e.g., in the form of a reduction in the *D* band peak intensity, we have decided to also present one deconvolved GO spectrum (of slightly lower fit goodness) including explicitly much weaker $D^{*}$ band instead of D1 (see Figure 2c). All results with both forms of deconvolution are presented in SM. An additional argument for using D1 instead of $D^{*}$ was the known strong and uncontrolled influence of the platelet edges as a form of defect, which is an unavoidable effect in a thick quasi-3D sample.

**Figure 3:**
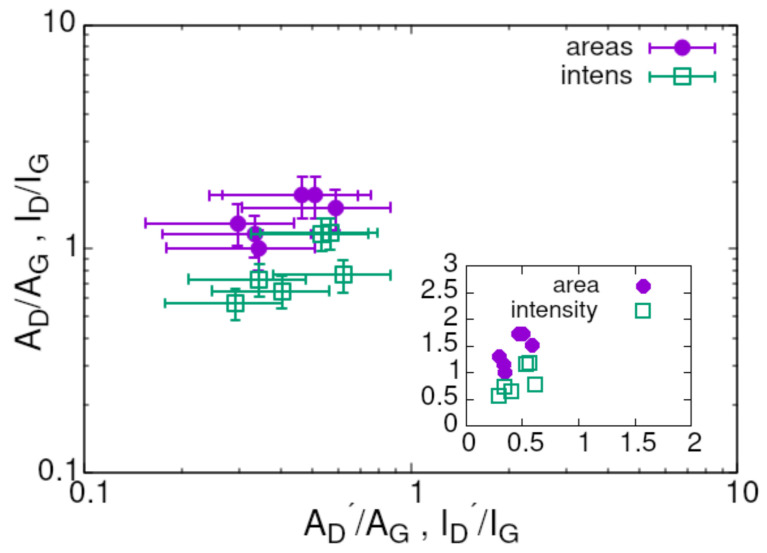
The dependence of $A_{D}$/$A_{G}$ vs. $A_{D^{\prime }}$/$A_{G}$ and for the corresponding peak intensities, i.e., $I_{D}$/$I_{G}$ vs. $I_{D^{\prime }}$/$I_{G}$ for all samples annealed at different temperatures. It is worth noting that the spectra deconvolved with the $D^{*}$ band were quasi-constant in these coordinates. The inset contains the same data but in linear coordinates.

Thus, one of the main questions arising in the context of applying the reduction process to the samples formed by the described method is the scale of the structural modifications caused by it. The ratio of intensities $I_{D}$/$I_{D^{\prime }}$ has been proposed as the indicator of the defect nature at a moderate disorder level [12]. A merging level of $D^{\prime }$ and *G* bands also points to some disorder level. Following the principle of using integrated intensities instead of the peak intensities, we applied and processed mainly the ratio $A_{D}$/$A_{D^{\prime }}$ but for the sake of completeness the intensities were used in some cases. The information about the behaviour of this ratio can be extracted from the dependence of $A_{D}$/$A_{G}$ on $A_{D^{\prime }}$/$A_{G}$ with the assumption of *G* band stability. This functional dependence is shown in Figure 3. The inset presents the same measurement points in the linear coordinates. The very similar character of the scatter for both the integrated and peak intensities supports the replacement idea. For graphene defected by oxidation, the value of $I_{D}$/$I_{D^{\prime }}$ of about 3.5 acted as an indicator for a limited disorder level with a dominance of the boundary defects [12]. The slope resulting from the linear regression applied to our data gave an $A_{D}$/$A_{D^{\prime }}∼$ 1.9 and let us conclude that disorder is limited and the boundary-like defects dominate in our samples. This conclusion should not be a big surprise, taking into account the character of morphology and internal structure of our samples illustrated in Figure 1b with many edges of the platelets.

Characteristically, all ratio values seen in Figure 3 concentrate around 1 and this implies that we are close to the transition area between low ($A_{D}$/$A_{G}$ ∼ 1/$L_{d}$) and high ($A_{D}$/$A_{G}$ ∼ 1/$L_{d}^{2}$) disorder levels. Using the formula $A_{D}$/$A_{G}$ = (102 ± 2)/$L_{d}^{2}$ derived in [52] for high defect concentration we can obtain the $L_{d}$ value, i.e., an average distance between the defects equal to 8.3 nm or alternatively 11 nm, if the peak intensity values were used.

**Figure 4:**
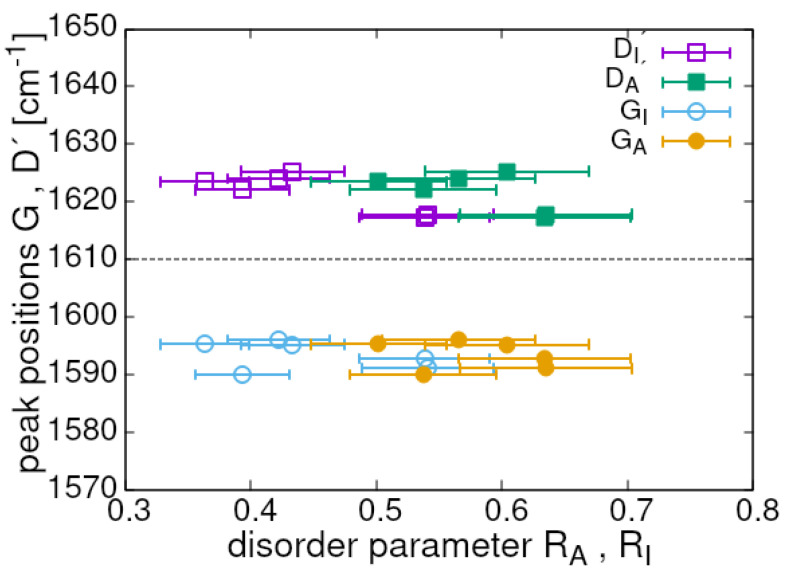
Dependence of the *G* and $D^{\prime }$ bands’ positions on the disorder degree. The band position was determined with an error lower than 1% and that could not be made to be visible.

Cuesta et al. [28] suggested a method of determining changes in the structural order of carbon materials based on functional dependence of the peak positions of the bands *D*, *G* and $D^{\prime }$ on the ratio $I_{D}$/$\left(I_{D}+I_{G}\right)$. From here on, we use the term *disorder parameter* (denoted as $R_{i}$ with *i* = A,I) to identify the ratio but essentially used its version containing the integrated intensities (band areas) [34,35,36,37,38]. It was stated in Ref. [28] that $I_{D}$/$(I_{D}+I_{G})>$ 60% indicates highly disordered solid carbon. All our thermally treated samples showed this parameter between 36% and 54% and when the formula $R_{A}\;$=$\;A_{D}/\left(A_{D}+A_{G}\right)$ was used, the disorder parameter was between 0.50 and 0.63, i.e., within a slightly shrunk range. Following the well-established procedures, we plotted the position of the peaks *G* and $D^{\prime }$ as a function of the disorder parameter. The result is visible in Figure 4 and the separation of the *G* and $D^{\prime }$ peaks’ positions stays nearly constant at the different applied temperatures. The position of both peaks should merge at what is classified as the beginning of high structural disorder. Importantly, the scatter character for both $R_{A}$ and $R_{I}$ is very similar. Extrapolation of our data suggests that the border for the disorder character is placed at $R_{I}≃$ 65% and equivalently for $R_{A}$ at about 70%.

**Figure 5:**
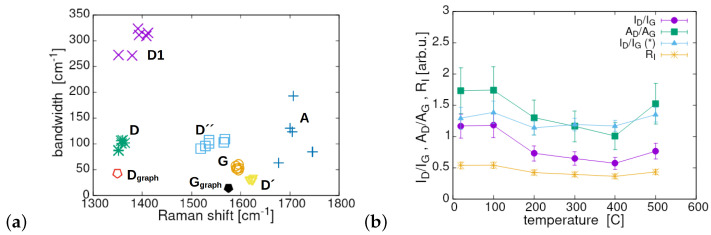
(**a**) Dependence of bandwidth on position of the relevant peak for the main deconvoluted bands. Each measurement point in a given scatter corresponds to a specific value of the annealing temperature. The lack of the error bars even in the cases where these could be applied is caused by keeping transparency of the data scatters. (**b**) The ratios of $I_{D}/I_{G}$, $A_{D}/A_{G}$ and the disorder parameter $R_{I}$ (defined in the intensity domain) vs. annealing temperature; the ratio $I_{D}/I_{G}$ with the deconvolution method applying $D^{*}$ is added for the sake of comparison.

The positions of the measurement points indicate that the material stays close to, but still below the disorder threshold, in accordance with the conclusion from Figure 4. The disorder level can be also qualitatively estimated from the plot presented in Figure 5a showing relation between the bandwidth and peak position of the specific components [39]. As the peak position and width of the main components could be influenced by the annealing temperature, all measurement points of a given band are shown in the plots. Thus, we obtained the range of changes initiated by the annealing process at different temperatures. Two single, low-laying points (the empty and filled pentagons) are given as references and illustrate the position and bandwidth (FWHM) of mono-crystalline graphite ($G_{graph}$) and minimally polycrystalline graphite ($D_{graph}$). The ideal mono-crystalline graphite does not show the *D* band. It is seen that the *G* band in our samples is very stable (hardly scattered) during the reduction and this justifies the analysis based on the data in Figure 3. The *D* band shows very limited scattering resulting dominantly from its width variations at nearly constant peak position. Obviously, the $D^{\prime }$ band shows a stability level similar to that of the *D* band or even better. This confirms a close relation between both. Other bands show different scattering levels and the strongest one belongs to *A*. The D1 band shows a slightly lower degree of scattering than *A* and its Raman shift moves during the reduction process down to 1360 cm^−1^. As a consequence, one can conclude that the investigated mlGO is still relatively stable and structurally a reasonably ordered material within the applied temperature range of the annealing process. Moreover, the data presented in Figure 5a regarding the bandwidth and position of the *G*, *D* and $D^{\prime }$ bands as well as the ratio $\Gamma_{D}/\Gamma_{G}$ (bandwidths) perfectly match the measurement results reported in [33] for the samples determined as nano-crystalline graphite and C/C composite. All available data collected for our samples suggest that the sample structures are definitely below, although not far, from what is seen as the high disorder threshold, independently of how we define it. The trends in variation of $I_{D}/I_{G}$, $A_{D}/A_{G}$ and the disorder parameter $R_{I}$ as a function of the annealing temperature are shown in Figure 5b. All show non-monotonic changes within a very limited range. To find more arguments and estimates related to the disorder scale we have also analysed the difference between the *D* band position at different annealing temperatures in relation to that of the slightly distorted polycrystalline graphite $D_{graph}$, again as a function of the disorder parameter. A very strong disorder is characterised by a linear increase in the parameter $|D-D_{graph}|$ vs. $R_{i}$, and clearly this was not the case with our samples.

**Figure 6:**
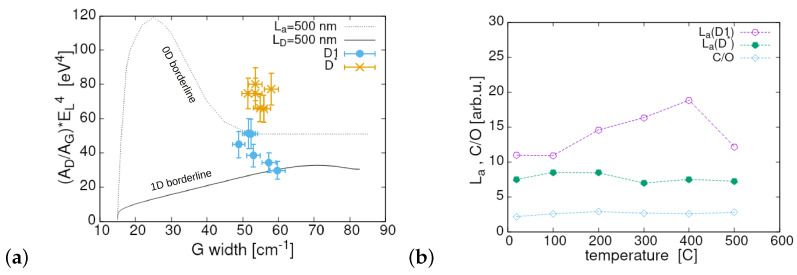
(**a**) The dependence between the theoretical parameter $\left(A_{D}/A_{G}\right)\cdot E_{L}^{4}$ and a width of the *G* band; all the measurement points corresponding to different annealing temperatures are shown and the dashed and solid lines determine theoretical borders corresponding to $L_{a}$ = 500 nm (dashed) and $L_{D}$ = 500 nm (solid), while the other parameter ($L_{D}$ or $L_{a}$, respectively) varies across the full range (0, 500 nm). The points marked by (×) correspond to the values obtained from deconvolution explicitly applying the band $D^{*}$. (**b**) The sizes of the crystallites for both deconvolution methods (labelled by D1 and $D^{*}$) together with the C/O ratios changes suggest the metastable character of the material.

The aforementioned idea of using integrated band intensity as a more adequate parameter for determining the scale of defects in the graphene-related materials [34,35,36,37,38] was based on the fact that the areal (integrated) band intensity is closely related to the probability of the given physical effect. Some new methodology offering disentangling the contribution from point and line defects in graphene-related materials was proposed in Ref. [37]. The authors prepared two differently parametrised plots called Raman diagrams and placed them in the coordinates with a dependence between $\left(A_{D}/A_{G}\right)\cdot E_{L}^{4}$ and a width of the *G* band (Figure 3 in Ref. [37]). Here, the parameter $E_{L}$ is the photon energy of the used radiation expressed in eV. The parametrised plot presented in [37] contains a very large set of the points plotted for different combinations of $L_{a}$ and $L_{D}$. These points constitute gussets of sort of a mesh enabling extraction of quite accurate combination ($L_{a}$, $L_{D}$) for given coordinates within the existing axes. We used the procedure proposed there as another reference to verify our estimates of $L_{a}$ (crystallite size understood as an extension (size) of the area with mono-crystalline character) and $L_{d}$ (an average distance between the defects). The result is presented in Figure 6a in the form of six scattered points corresponding to different reduction temperatures. Interestingly, all points are between two limiting curves of the Raman diagram reproducing the structure with pure point defects (dashed) and pure line defects (solid). Thus, it defines the space covering samples with 0-D and 1-D defects. On the other hand, the authors of Ref. [37] indicated the area for cautiousness due to multilayer structure or strain not taken into account in calculating the Raman diagram’s curves. However, the results still should span the full range from an ideal structure to full disorder. The coordinates defining the centre of our measurement points collection ($\Gamma_{G}$,($A_{D}$/$A_{G}$)$\cdot E_{L}^{4}$) were determined as (53.8 cm^−1^, 41.6 eV^4^). Having located these coordinates within the “Raman diagram” of [37] we were able to evaluate the values of $L_{a}$ and $L_{d}$. These were in the range of 13.1 nm and 8.4 nm, respectively. The first one reasonably corresponds to the estimate of $L_{a}$ (an average value of 14 nm) by using the formula $L_{a}$ = 560/[($A_{D}$/$A_{G})\cdot E_{L}^{4}$] given in Refs. [35,53] and dedicated rather to thin and quite ordered materials. It is worth noting that the plot in Figure 6a distinctly demonstrates the consequences of applying two different methods in the deconvolution process. The points marked with (x) correspond to the deconvolution with the $D^{*}$ band. They suggest a similar $L_{a}$ value of ≃13 nm but a significantly smaller $L_{d}$, located in the range between 2 and 3 nm. The functional dependence of $L_{a}$ on the annealing temperature for both variants of the deconvolution method together with the temperature dependence of the C/O ratio are seen in Figure 6b. The average crystallite size ($L_{a}$) increased monotonically from 11 nm at RT to a value of ≃18 nm for the annealing temperature of 400 °C, then to fall to 12 nm at 500 °C, while the same parameter for deconvolution with the $D^{*}$ band fluctuated around 8.5 nm (see Figure 6a). The plot of the C/O ratio was included to indicate the small variations in the structural and chemical parameters within the reduction process and support the claim that such a material has a metastable character [17].

**Figure 7:**
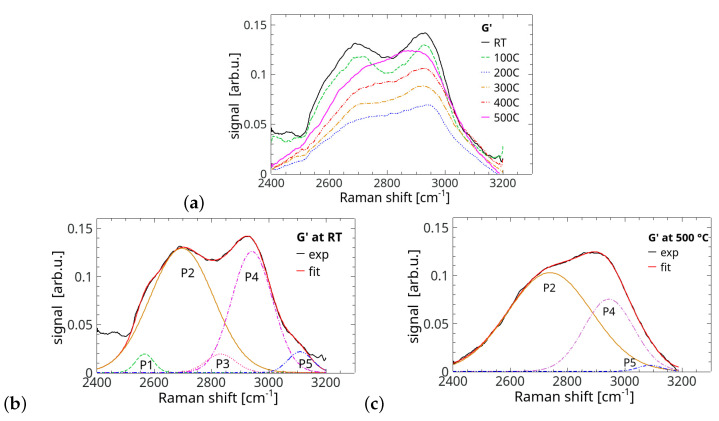
(**a**) Raman spectra of the $G^{\prime }$ band with the varying annealing temperature; (**b**,**c**) deconvolved Raman spectra of $G^{\prime }$ band recorded, respectively, for the sample without annealing (RT) and for that annealed at 500 °C.

The $G^{\prime }$ (known also as 2*D*) band is considered as a source of information on the stacking order in graphene-related materials as this part of the spectrum is very sensitive to structural changes along the *c*-axis [54] and electron mobility. The label $G^{\prime }$ has a quite long tradition and has been used here to distinguish the whole second-order band from its 2*D* component being exactly the overtone of the band *D*. Changes of the full $G^{\prime }$ band with the annealing temperature and its deconvolved versions at RT and after annealing at 500 °C are presented in Figure 7a–c. Again, the form of $G^{\prime }$ at RT resembles that of few-layer wrinkled graphene (flwG) derived from graphite oxide [16] as well as that presented in [28] for natural microcrystalline graphite with a determined disorder parameter $R_{I}$ of about 69%. Interestingly, the $G^{\prime }$ band has the lowest intensity after a reduction of 200 °C, i.e., directly after the assumed removal of water. This effect points to the maximum defect concentration [12].

The plots in Figure 7b show that a reasonable fit of the second-order spectra for the RT sample required five components of different intensities placed roughly at 2567 cm^−1^ (P1), 2697 cm^−1^ (P2), 2833 cm^−1^ (P3), 2942 cm^−1^ (P4) and 3110 cm^−1^ (P5). It should be noted that the labels $P_{1}$–$P_{5}$ are selected arbitrarily and should not be confused with those applied in the consideration of the double-resonance mechanism involved in creation of the $G^{\prime }$ band. Here, the low-frequency side of the second-order spectrum was influenced by the presence of the *B* band discussed earlier and that was ignored during the fitting. Only three of the contributing bands, namely P2, P4 and P5, “survived” the reduction process (Figure 7c). P2 and P4 are usually ascribed to the modes D+D and D+G, respectively. Both bands at approximately 2700 cm^−1^ and 2900 cm^−1^ appear in noticeably disordered carbon materials, while the highly graphitised (highly ordered) carbon materials do not show the last band at all [28]. The picture of the deconvoluted band $G^{\prime }$ after annealing our sample at 500 °C is typical for a carbon-based material disordered to some extent, and the band with the peak at 2944 cm^−1^ being a disorder signature was strongly diminished in the reduction process. This band was accompanied by the typically strongest one positioned at 2738 cm^−1^ (2D). Such a behaviour and the signal peak level (normalised to $G_{app}$) suggest an increase in the ordering level caused by the reduction process and the structure clearly tends towards a turbostratic character of the disorder normally characterised by a single broad peak. Thus, the result supports the conclusion that the annealing process at moderate temperatures and in a vacuum definitely improves the material’s structural order but it still shows defect-activated bands of noticeable intensity. It is also worth noting that the ratio of intensities $I_{2D}$ and $I_{G}$ is commonly treated as an indicator of the number of planes for flG and flGO. The reduction process should not change this value but it did. This change is easily seen from the presented deconvolved bands as all data were normalised to the intensity of $G_{app}$. It varies between 0.30 for the RT sample and 0.17 for the sample annealed at 500 °C. This shows the difficulty in the problem of very thick samples due to disorder along the *c*-axis. Interestingly, the other deconvolution method using the $D^{*}$ band gives a value of $I_{2D}$/$I_{G}$ equal for both cases. However, the absolute of this ratio is higher and does not correspond to more than 1000 layers present in our samples. This stresses the difficulty of the problem.

## 4. Summary and Conclusions

Samples of multilayer graphene oxide (GO paper) were prepared using a very simple method relying on short-time embedding a standard copper mesh in a water suspension of GO platelets followed by drying in environmental air, then undergoing an annealing process (heating for 1/2 h in vacuum). They were then analysed using micro-Raman spectroscopy. The process delivered a set of solid self-standing (without any substrate) samples and our main interest was focussed on the influence of such a procedure on the material structure. The same samples showed some unusual electronic properties in the area of σ-resonances when investigated with the NEXAFS and XPS methods [18].

Both the first- and second-order spectra allowed for the conclusion that the samples represent a significant disorder level that is still below the value considered the border line of a very high/full disorder. The estimated average crystallite size $L_{a}$ = 11–13 nm and an average distance between the defects $L_{d}$ of ≃8 nm (alternatively 2-3 nm for different deconvolving procedure) were found for samples prepared in the described way. The material structure properties defined by the main GO membrane spectral parameters (position and bandwidth of the *D* and *G* bands) were relatively stable during the reduction process at temperatures up to 500 °C. The samples confirmed the effect of metastability by the stable C/O ratio and influence of the occurring chemical processes initiated by water. Water delivers hydrogen and oxygen and both are important due to supporting creation of the hydrogen bonds, which change the relation between the basal planes and the molecular functionals. The other important conclusion is the confirmation of the ordering effect of the moderate annealing process and the data suggest that higher temperatures strengthen the impact.

The unexpected bands observed in the Raman spectra were related to the intercalated water and that, we think, initiated a rich spectrum of chemical reactions reflected in the existence of new features in the spectra. One of the new features, denoted as *A*, was ascribed to carbonyl functionals in the carbonyl-containing reaction products as carboxylic acids, esters or ketones. However, the open question regarding the contribution of the electronic excitation and double resonance to the same spectral region still exists. The other one (*B*), strongly declined by annealing at the temperature below 200 °C, could not be clearly identified due to lack of specific data allowing for unambiguous identification. We think that it was caused by the intercalated water together with the assumed presence of sp-hybridised carbon chains. The evident influence of such a reduction process on structural rearrangement along the material *c*-axis was distinctly visible in the modification of the second-order spectra ($G^{\prime }$) where deconvolved bands tended towards the single one positioned at ∼2740 cm^−1^, which is the feature characterising the turbostratic stacking order. The D1 band was also strongly reduced and down-shifted by the annealing process.

## Figures and Tables

**Table 1:** Table with the values of the most important parameters considered in the manuscript when compared with examples taken from some other publications.

Production Method	T [°C]	$D_{\mathrm{pos}}/\mathrm{FWHM}$	$G_{\mathrm{pos}}$/FWHM	$I_{D}/I_{G}$	C/O	Ref.
modif. Hummers/sediment.	RT	1355/102	1589/52	1.2 (1.3) ^1^	2.2	[18]
	500	1364/112	1592/49	0.77 (1.35)	2.8	[18]
modif. Hummers/TPD	RT	1364	1583	0.9	2.3	[21]
	127	1364	1583/broad. ^2^	1.2	5.3	[21]
modif. Hummers	RT	1337/119	1594/82	0.99		[22]
commerc. carbon powder	RT	1343–1348	1590–1598	0.93		[23]
	140–200	1343–1348	1590–1598	1		[23]

^1^ Value in bracket from deconvolution with $D^{*}$. ^2^ Broadened but without specific value.

## Data Availability

The available data can be obtained on request.

## Abbreviations

The following abbreviations are used in this manuscript:

GO	graphene oxide
slGO	single-layer graphene oxide
flGO	few-layers graphene oxide
